# Liquid–liquid phase separation and conformational strains of *α*-Synuclein: implications for Parkinson’s disease pathogenesis

**DOI:** 10.3389/fnmol.2024.1494218

**Published:** 2024-10-23

**Authors:** Eva D. Ruiz-Ortega, Anna Wilkaniec, Agata Adamczyk

**Affiliations:** Department of Cellular Signalling, Mossakowski Medical Research Institute, Polish Academy of Sciences, Warsaw, Poland

**Keywords:** Parkinson’s disease, alpha-synuclein, liquid–liquid phase separation, protein aggregation, conformational strains, cellular stress, mitochondria

## Abstract

Parkinson’s disease (PD) and other synucleinopathies are characterized by the aggregation and deposition of alpha-synuclein (*α*-syn) in brain cells, forming insoluble inclusions such as Lewy bodies (LBs) and Lewy neurites (LNs). The aggregation of *α*-syn is a complex process involving the structural conversion from its native random coil to well-defined secondary structures rich in *β*-sheets, forming amyloid-like fibrils. Evidence suggests that intermediate species of *α*-syn aggregates formed during this conversion are responsible for cell death. However, the molecular events involved in *α*-syn aggregation and its relationship with disease onset and progression remain not fully elucidated. Additionally, the clinical and pathological heterogeneity observed in various synucleinopathies has been highlighted. Liquid–liquid phase separation (LLPS) and condensate formation have been proposed as alternative mechanisms that could underpin *α*-syn pathology and contribute to the heterogeneity seen in synucleinopathies. This review focuses on the role of the cellular environment in *α*-syn conformational rearrangement, which may lead to pathology and the existence of different *α*-syn conformational strains with varying toxicity patterns. The discussion will include cellular stress, abnormal LLPS formation, and the potential role of LLPS in *α*-syn pathology.

## 1 Introduction

Parkinson’s disease (PD) is recognized as the second most common neurodegenerative disease (Du et al., 2020; Serratos et al., 2022) and is part of a heterogeneous group of disorders, so-called synucleinopathies, that include dementia with Lewy bodies (DLB), multiple system atrophy (MSA) (So and Watts, 2023) among others. As referred to by their name, a common hallmark of these disorders is the abnormal accumulation of *α*-synuclein (*α*-syn) (encoded by the SNCA gene), in neurons and neurites, glia, and presynaptic terminals (Kramer and Schulz-Schaeffer, 2007). In these, *α*-syn accumulates as inclusions in the form of Lewy bodies (LB) and Lewy neurites (LN) collectively referred to as Lewy pathology (LP) (Spillantini et al., 1997; Goedert et al., 2017; Killinger et al., 2022). Whereas in MSA, the inclusions found in oligodendrocytes are called glial cell inclusions (GCIs) (Kramer and Schulz-Schaeffer, 2007).

Identifying genetic defects related to the *α*-syn gene, such as mutations and copy number variations in families with hereditary disorders, reinforces its significance (SNCA Gene: MedlinePlus Genetics, 2021). Consequently, it is well-established that *α*-syn is the central protein involved in the pathology of several neurodegenerative diseases, including PD and other synucleinopathies (Lee and Trojanowski, 2006; Poulopoulos et al., 2012; Goedert, 2015; Ugalde et al., 2016; Cremades et al., 2017; Meade et al., 2019; Du et al., 2020). The presence of *α*-syn inclusions is a defining feature of these conditions, and research has primarily focused on the pathological (aggregated) forms of *α*-syn as the main factor in neurodegeneration, a conclusion that remains well-supported today. Despite this, the fundamental aspects that concern *α*-syn and its role in the underlying pathogenesis of PD and other synucleinopathies remain unknown, and the details about how *α*-syn mediates toxicity are not fully elucidated. Furthermore, growing evidence indicates a significant variability in clinical and pathological manifestations between different synucleinopathies, and, more strikingly, this variability also exists within the same disease entity (So and Watts, 2023). To reveal what could be a potential reason for such inconsistency among a group of disorders caused by the same protein, we will collect the current knowledge with particular attention to the environmental factors and conditions that may contribute to *α*-syn structural re-arrangement, leading to the formation of various strains of *α*-syn aggregates, with differential toxicity patterns. Furthermore, we will also focus on exploring the abnormal formation of a phenomenon called liquid–liquid phase separation (LLPS), cellular stress leading to chronic neuroinflammation and immune dysregulation, as well as the potential role of LLPS in *α*-syn pathology. We will also aim to explore how the variability of the *α*-syn aggregate might be related to pathological manifestations at the cellular level. This information is vital to understanding the current experimental models and ongoing development of disease-modifying and diagnostic tools.

## 2 *α*-Syn aggregation and formation of multiple conformational strains

Numerous studies indicate that the hallmark pathological features of synucleinopathies primarily arise from the abnormal folding of *α*-syn and its subsequent aggregation into intracellular inclusions (Grosso Jasutkar et al., 2022). This process contributes to the formation of potentially toxic *α*-syn species, which may also have the capacity to propagate throughout the brain in a prion-like neuron-to-neuron manner (Jan et al., 2021). Consequently, understanding the various structural modifications of *α*-syn is crucial for elucidating the onset and progression of these diseases. Under abnormal conditions, native monomeric and soluble *α*-syn undergoes a conformational shift from a random coil to a *β*-sheet structure. This transition leads to the formation of non-filamentous *α*-syn species that are on-pathway to further polymerization and aggregation, resulting in oligomers and protofibrils. The accumulation of these species promotes self-assembly and subsequent aggregation, ultimately leading to the formation of highly ordered, cross-β-sheet insoluble fibrils, as reviewed by Goedert (2015); Cremades and Dobson (2018); Du et al. (2020), and So and Watts (2023).The insoluble fibrils, together with crowded organelle components such as lipids, vesicular structures, damaged lysosomes, mitochondria, and other proteins within dying neurons eventually form the characteristic inclusions, such as Lewy pathology (LP) (Cremades et al., 2012; Shahmoradian et al., 2019; Fanning et al., 2020; Scheiblich et al., 2021).

**Figure 1:**
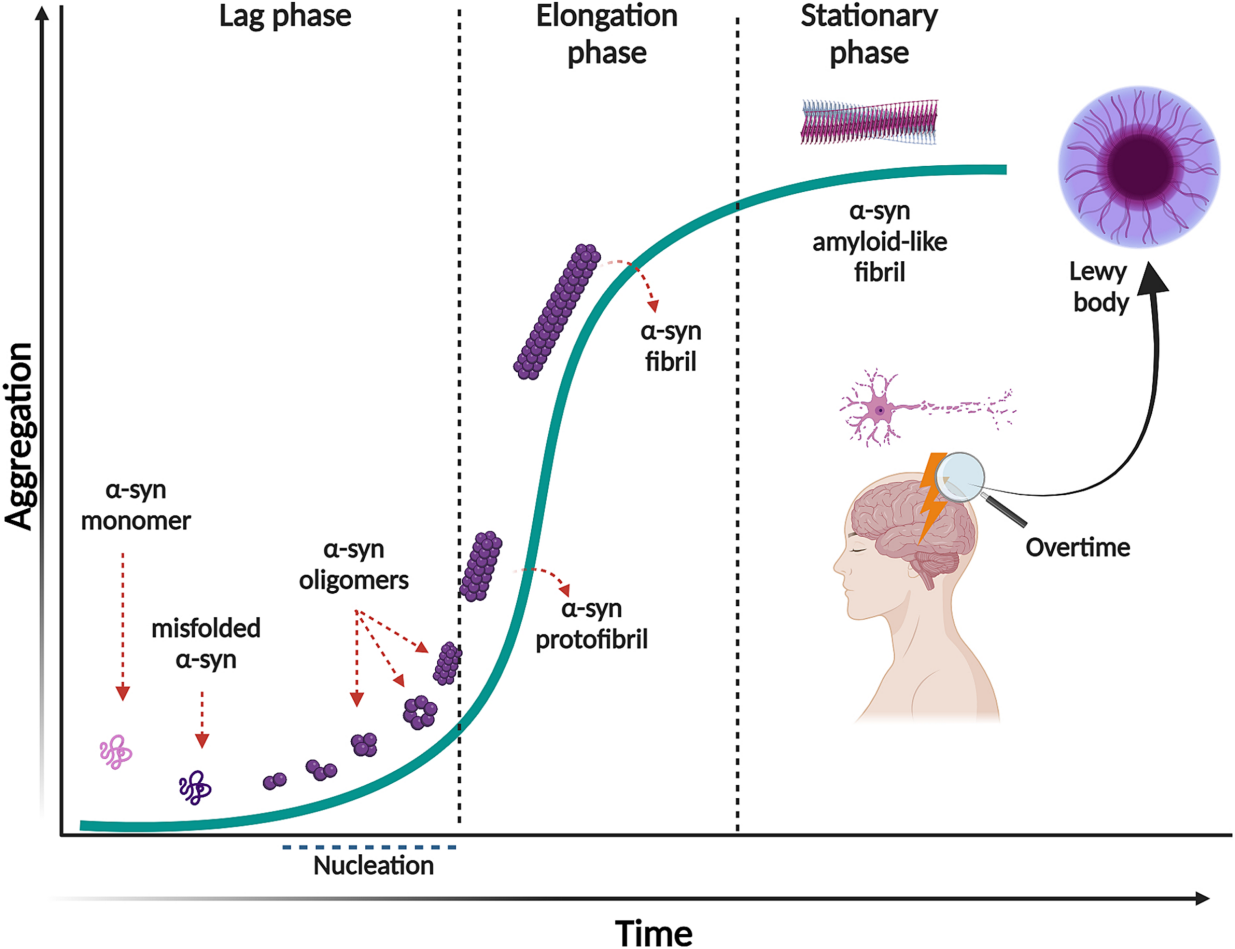
Nucleation-dependent polymerization model of *α*-syn aggregation. This process consists of three separate phases: a lag phase in which native monomeric *α*-syn change its conformation, misfold and form the aggregation competent specie, nuclei. This phase is thermodynamically unfavorable, and polymerization occurs slowly. In the elongation phase, *α*-syn nuclei rapidly convert into oligomers, protofibrils and fibrils. In the stationary phase most of the soluble *α*-syn has been converted into insoluble amyloid-like fibrils, reaching saturation. Created with BioRender.com.

The aggregation of *α*-syn follows a mechanism of nucleation-dependent polymerization pathway, with sigmoidal growth kinetics (Wood et al., 1999). This process consists of three separate phases (Figure 1). First, the lag phase where events are mainly thermodynamically unfavorable due to a high free energy barrier for abnormal aggregation. During this phase, native soluble and monomeric *α*-syn slowly polymerizes to form aggregation-competent species called nuclei. When *α*-syn nuclei reach a critical size, it acts as a seed that nucleates the aggregation pathway. At this stage, intermolecular interactions within the *α*-syn assemblies promote stability and prevent their dissolution. The second phase is the elongation or exponential growth in which the *α*-syn nuclei rapidly convert into oligomers, protofibrils, and fibrils by attaching monomeric *α*-syn onto them. Interestingly, the fibril growth involves different mechanisms, such as surface-mediated secondary nucleation which is templated by already formed fibrils catalyzing the aggregation process; fibril fragmentation, a process that generates multiple short fibrils that can template misfolding and assembly, as well as elongation (Gaspar et al., 2017). The third phase, the stationary phase, is where most of the soluble *α*-syn has been converted into insoluble amyloid-like fibrils and a steady state equilibrium is achieved between the fibrils and the monomers (Gaspar et al., 2017). It is important to mention that the initial misfolding and transformation of *α*-syn is poorly understood. However, it is quite established that the structural transition from random coil to *β*-sheet-rich insoluble fibrils relates to pathology and can happen in a sequence of events or through ramifications of the classical aggregation pathway, as reviewed in (Miraglia et al., 2018).

**Figure 2:**
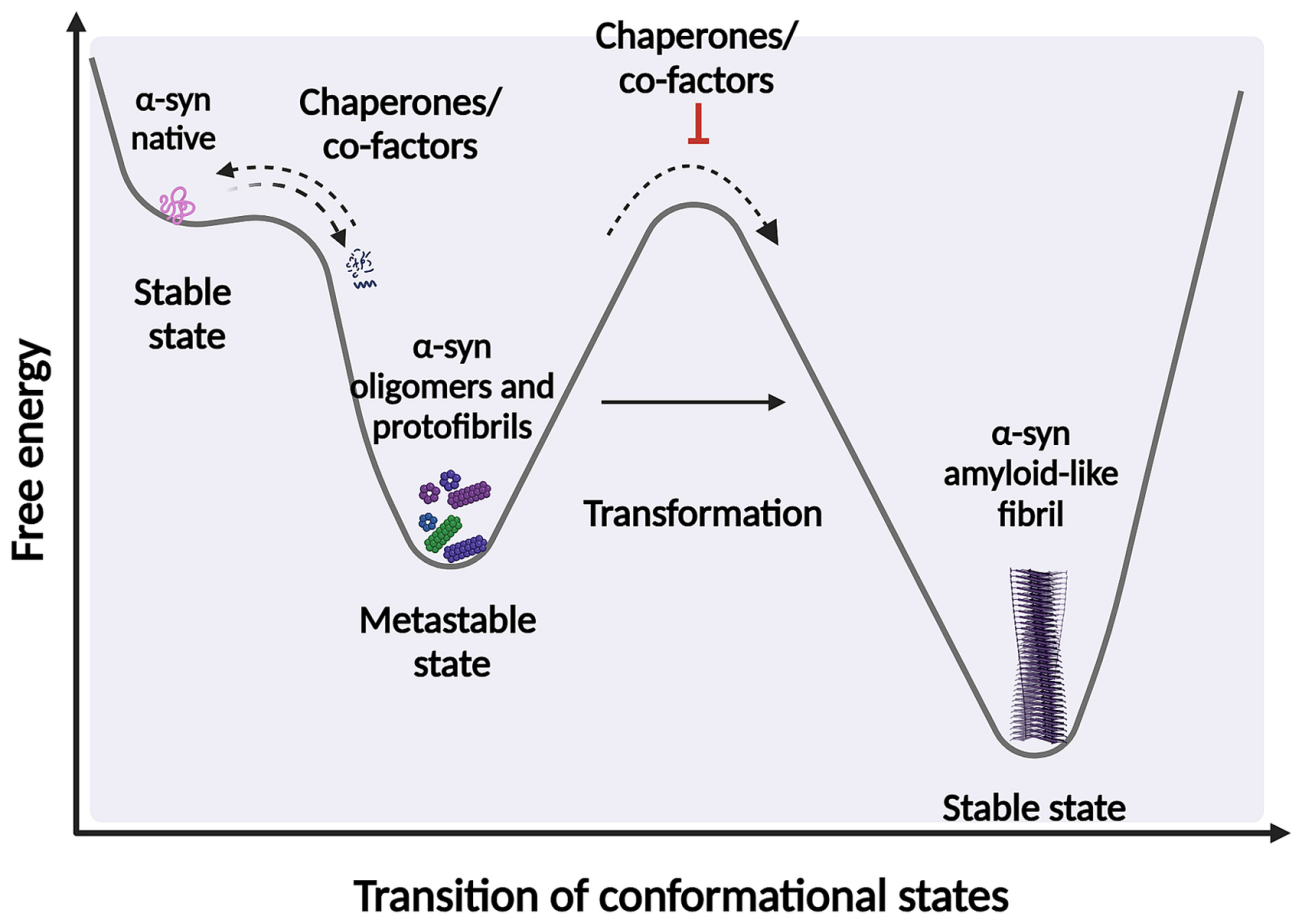
Scheme representing the structural transition during protein-folding. Under certain circumstances, intermediate structural conformation can escape from native folding and become kinetically trapped. These intermediate conformations require overcome an energy barrier to achieve a favorable downhill path to form highly ordered stable of species. *α*-syn protein transformations can be accelerated by factors and co-factors, such as posttranslational modifications (PTM) and chaperone by adding energy to significantly reduce the kinetic barrier. Fibrillary aggregation can be triggered after destabilization of the native state (partially folded states) which can also be prevented or reversed by molecular chaperones. Created with BioRender.com.

It is widely accepted that intermediates, both non-filamentous species such as oligomers, and filamentous intermediates, known as protofibrils are the toxic species that lead to neuronal death (Sharon et al., 2003; Karpinar et al., 2009; Winner et al., 2012; Cremades et al., 2017; Cremades and Dobson, 2018; Vicario et al., 2018; Ugalde et al., 2019; Meade et al., 2019; Lövestam et al., 2021; Emin et al., 2022; Gadhe et al., 2022). These species exist in a thermodynamically metastable environment where they can be kinetically trapped (with a minimum local free energy) and are prone to transform into different structural conformations (Ghosh and Ranjan, 2020) (Figure 2). However, they must overcome an energy barrier to achieve a stable and highly ordered conformation, and this transformation does not occur spontaneously depending on various environmental conditions, such as *α*-syn posttranslational modifications (PTM) and mutations, alternative splicing of the coding exons, chaperone proteins, lipids, and other factors. These conditions help to reduce the kinetic barrier, accelerating *α*-syn aggregation (Vidović and Rikalovic, 2022). Additionally, during the formation of different *α*-syn conformational states, only those sufficiently stable will persist and grow to form filaments (Peelaerts et al., 2018). This metastable character is the reason why these *α*-syn intermediate species can quickly change their morphological and biochemical properties (surface properties), forming a short-lived transient population that coexists as heterogeneous conformations (structurally unique aggregates), more commonly called strains. The existence of this short-lived and transient population also explains why their structural characterization is extremely complicated (Meade et al., 2019; Du et al., 2020; Han et al., 2024).

Intermediate species such as *α*-syn oligomers have been described as soluble (as opposed to fibrils) although they possess a varying degree of hydrophobicity (Chen et al., 2015; Lee et al., 2018). Some oligomers can have different sizes, structures, and morphologies (Emin et al., 2022), for instance spherical, cylindrical or annular, chain-shaped, and also tubular (Miraglia et al., 2018). Some oligomers have an intermediate *β*-sheet content between monomers and fibrils (Hong et al., 2008; Chen et al., 2015). In addition, some of these can adopt a β-sheet structure with an antiparallel arrangement (stabilized oligomers-kinetically trapped) (Chen et al., 2015) that differs from the classical β-sheet geometry. This could influence oligomer toxicity (Celej et al., 2012) or monomer binding capacity, as species with antiparallel arrangements are less stable and less efficient at elongating (Chen et al., 2015). Other evidence supports the hypothesis that these heterogeneous conformations show toxic disparity depending on the different structures [reviewed by Meade et al. (2019); Du et al. (2020), So and Watts (2023), and Han et al. (2024)]. For instance, ultrastructural analysis has shown that oligomers with cylindrical or donut-shaped conformation may interact favorably with hydrophobic membranes contributing to toxicity (Chen et al., 2015; Meade et al., 2019). Although small oligomers are kinetically unstable and can dissociate into monomeric forms (Chen et al., 2015; Meade et al., 2019), some evidence suggests that this less stable form of *α*-syn induces greater toxicity when compared to other species (Chen et al., 2015; Meade et al., 2019). Conversely, fibrillar forms of *α*-syn, that are recognized to be kinetically more stable compared to oligomeric and intermediate species (Chen et al., 2015; Bisi et al., 2021), can sequester toxic oligomers and eventually convert them into more thermodynamically stable fibrils, leading to potential beneficial outcomes for cells [as reviewed Gadhe et al. (2022)].

Although *α*-syn fibrils are generally considered less toxic than their intermediate species, their pathological role remains significant [as recently reviewed by Bigi et al. (2023)]. For instance, small *α*-syn fibrils are known to bind efficiently to cell surface receptors, such as heparin sulfate proteoglycans (Holmes et al., 2013) and possess a strong capacity to disrupt cellular membranes and other processes, including mitochondrial function [reviewed by Domingues et al. (2022)]. Moreover, *α*-syn fibrils are highly prone to fragmentation within cells, leading to the formation of numerous small structures. These fragments act as potent seeds, facilitating the propagation of toxic, misfolded *α*-syn aggregates to neighboring brain regions (Luk et al., 2012; Masuda-Suzukake et al., 2013; Volpicelli-Daley et al., 2011, 2014; Thakur et al., 2017; Cascella et al., 2021; Bigi et al., 2021, 2022). These findings underscore the pivotal role of prion-like behavior and propagation in the clinical manifestations of human diseases [as reviewed by Bigi et al. (2023) and Gadhe et al. (2022)]. Determination of *α*-syn conformational strains has been linked more frequently to fibrils, as small oligomeric species lack ordered structures and stable conformations. Therefore, studying their properties using common methods, such as cryo-electron microscopy (cryo-EM) (Schweighauser et al., 2020; Yang et al., 2022) or solid-state NMR spectroscopy (Heise et al., 2005; Gath et al., 2014; Tuttle et al., 2016; Li et al., 2018a; Li et al., 2018b; Cao et al., 2019; Kollmer et al., 2019) is complicated. However, *α*-syn fibrillar forms have been well characterized based on diameter, proto-filament packing, number of twists and side chain interactions, and relative resistance to proteolytic enzymes (Gath et al., 2014; Li et al., 2018b; Guerrero-Ferreira et al., 2019).

**Figure 3:**
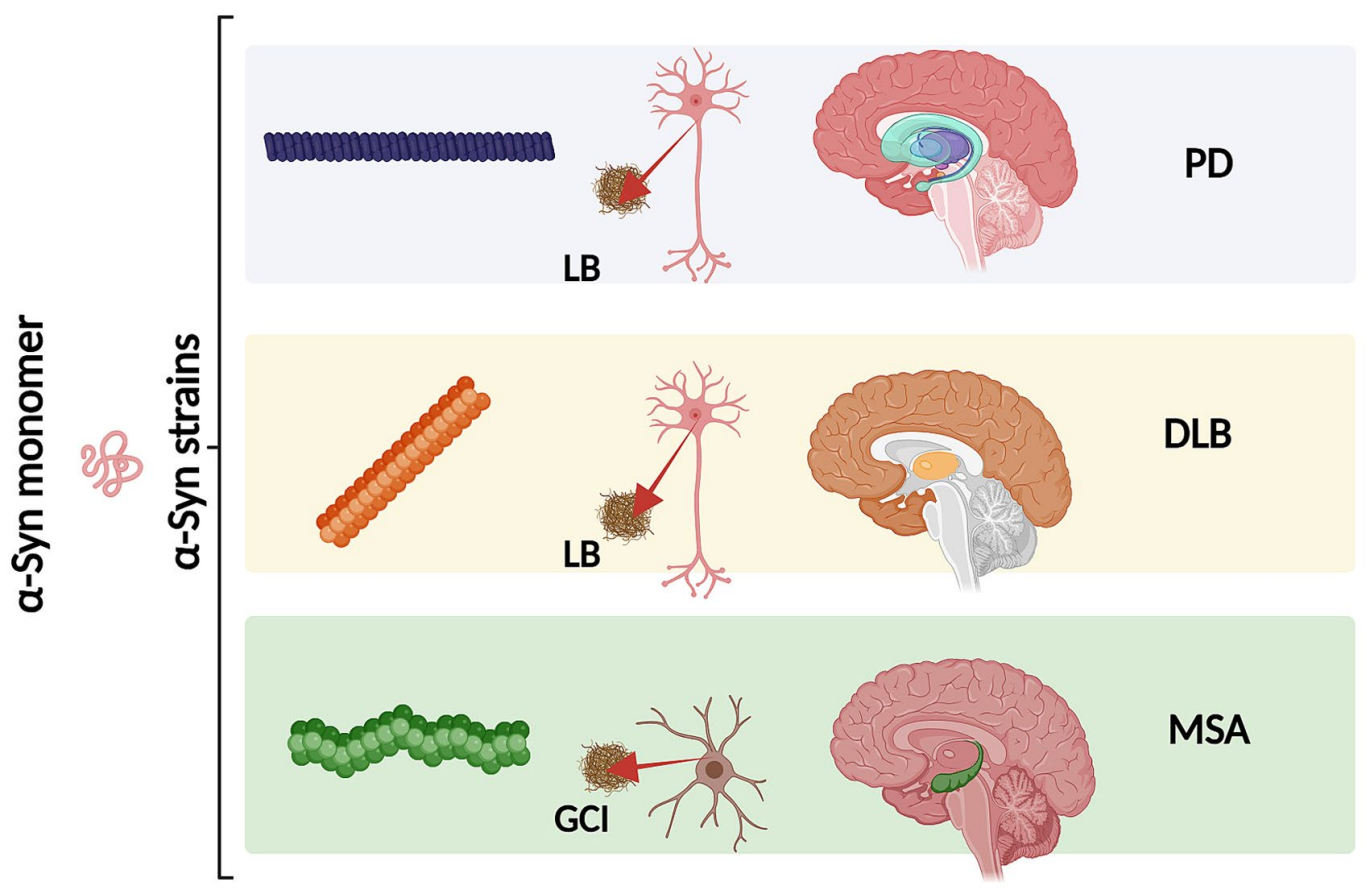
Schematic representation of different *α*-syn conformational strains in classical *α*-synucleinopathies (PD, DLB and MSA). Conformational strains arise from a different folding and aggregation of the same monomeric protein precursor which in turn might lead to pathological variability. The *α*-syn strains might be associated both with the cell type and type of *α*-syn inclusion. As we as with the neurodegeneration pattern and disease severity. Created with BioRender.com

An increasing number of observations report the presence of different conformational strains of endogenous *α*-syn in postmortem brains of patients suffering from PD, MSA, and DLB [as reviewed in Peelaerts et al. (2018) and more recently in Wojewska et al. (2023)]. This leads to the hypothesis that *α*-syn may adopt different disease-causing conformations that determine the destiny of pathology and may manifest in individual clinical phenotypes (Figure 3) (Peelaerts et al., 2015; Peng et al., 2018b; Peng et al., 2018a; Rey et al., 2019; Lau et al., 2020; Shahnawaz et al., 2020; Holec et al., 2022; Khedmatgozar et al., 2024). Supporting this hypothesis, it has been reported that various conformations of *α*-syn inclusions generated *in vitro* (referred by the authors as fibrils, ribbons, fibrils-91, fibrils-65, and fibrils-110) lead to differential binding and clustering in the neuronal membrane (Shrivastava et al., 2020). This results in distinct patterns of synaptic receptor redistribution in primary neuronal cultures and organotypic cultures of hippocampal slices from wild-type mice (Shrivastava et al., 2020). Furthermore, conformation-dependent seeding activity and alteration in neuronal network activity after seeded *α*-syn aggregation were also observed, affecting neuronal homeostasis through the redistribution of synaptic proteins (Shrivastava et al., 2020). In MSA patients, immuno-EM and cryo-EM analysis revealed the existence of two distinct *α*-syn filament types, characterized by the asymmetric packing of the two protofilaments that compose them, in addition to an extended N-terminal arm and a compact C-terminal body. More interestingly, both structures show a central cavity that incorporates non-protein molecules, suggesting the involvement of various factors and co-factors during their formation. The two reported MSA aggregates are distinct from those isolated from the brains of PD, Parkinson’s disease dementia (PDD), and DLB patients, where a single untwisted protofilament typically forms fibrillar inclusions (Tarutani et al., 2018; Schweighauser et al., 2020). However, a subpopulation of twisted aggregates has also been described in DLB (Yang et al., 2022). These MSA structures also differ from those obtained *in vitro* using recombinant *α*-syn (Schweighauser et al., 2020). Aggregates from PD, PDD, and DLB patients have a unique structural feature known as the Lewy fold, characterized by a salt bridge between residues E35 and K80. In contrast, MSA structures exhibit a salt bridge between residues E46 and K80 (So and Watts, 2023).

As different structural features between distinct conformations, some biochemical characteristics could also define these strains among human synucleinopathies. For instance, the exposure of recognition sites of proteins to enzymatic digestion agents generates various proteolytic fragments. Differences in protease resistance (PK and TL digestion) have been reported in *α*-syn fibrils isolated from LB (LB-*α*-syn) and MSA brains (GCI-*α*-syn), the latter being the more resistant to protease digestion (Peng et al., 2018a). A recent study reported that *α*-syn fibrils isolated from MSA brains gradually disappear after PK treatment but are not digested into small fragments as seen in filaments from PDD and DLB. This suggests that *α*-syn fibrils from MSA brains are more easily digestible and exhibit characteristic biochemical instability (Lee et al., 2024). This is important because greater resistance could be related to a slower degradation rate of *α*-syn species, potentially leading to a more efficient propagation of *α*-syn pathology in MSA (So and Watts, 2023). These propagation properties are related to the high ability of *α*-syn species to act as seeds that can be taken up by cells and template the misfolding of monomeric *α*-syn (So and Watts, 2023). It has been demonstrated that MSA-related aggregates are more potent than Lewy body inclusions (LB-*α*-syn) in seeding *α*-syn aggregation and maintain their high seeding activity when propagated in neurons (Peng et al., 2018a). This is in line with other findings showing that *α*-syn inclusions from MSA brains are less resistant to denaturation by detergents such as guanidine hydrochloride (GdnHCl) (Lau et al., 2020; Martinez-Valbuena et al., 2022; Lee et al., 2024) and sodium dodecyl sulfate (SDS) (Campbell et al., 2001) compared to *α*-syn fibrils from both DLB (Lau et al., 2020; Martinez-Valbuena et al., 2022; Lee et al., 2024) and PD (Lau et al., 2020; Martinez-Valbuena et al., 2022). Detergents such as GdnHCl, can act as chaotropic agents and denature through interactions with the polar regions of *α*-syn. Therefore, greater resistance to strong detergents suggests greater stability or less polar interactions within the protein. This is supported by previous observations in which upon exposure of human samples to SDS, more insoluble species are generated in DLB and PD than in MSA samples (Campbell et al., 2001) *α*-syn fibrils from MSA are smaller and more fragile as well as both more compact (in terms of packaging) and prone to recruit native *α*-syn molecules to form aggregates and filaments (seeding activity) compared to other filaments (So and Watts, 2023), such as LB human brains (Prusiner et al., 2015; Woerman et al., 2015; Peng et al., 2018b; Yamasaki et al., 2019). MSA fibrils are more easily fragmented by detergents (due to less conformational stability). Fragmentation of *α*-syn fibrils produces newly formed nuclei that can then catalyze additional misfolding and aggregation of monomeric *α*-syn, resulting in an exponential amplification of the original seed. This in turn may increase their propensity to seed and spread its aggregation (Peng et al., 2018a; Peng et al., 2018b). This is supported by the more rapid progression as well as the poor prognosis of MSA (Foubert-Samier et al., 2020; So and Watts, 2023; Wojewska et al., 2023). These findings are consistent with the notion that different *α*-syn strains underlie PD and MSA (Yamasaki et al., 2019) and highlight the importance of conformational stability as a possible explanation for the more rapid rates of disease progression in MSA compared to PD (So and Watts, 2023). This also supports the hypothesis that both the onset and progression of the disease are specific to each strain (as recently reviewed by Khedmatgozar et al., 2024). These stages are determined by the amount of energy required for the *α*-syn monomer to transform into a certain conformation which varies across different pathologies, such as MSA and PD (Khedmatgozar et al., 2024). The *α*-syn protein misfolding cyclic amplification (PMCA) method, known as *α*-syn-PMCA assay (Shahnawaz et al., 2020) or *α*-syn-RT-QuIC assay (Fairfoul et al., 2016; Groveman et al., 2018; Nakagaki et al., 2021) have been used to discriminate between cerebrospinal fluid samples from patients diagnosed with PD and MSA and is based on the seeding-nucleation mechanism to amplify small amounts of *α*-syn oligomers to facilitate their detection by other biological methods. The use of *α*-syn-PMCA assay has contributed to generating additional evidence indicating that *α*-syn aggregates associated with PD and MSA correspond to different conformational strains of *α*-syn (Shahnawaz et al., 2020). These studies highlight that the various forms of *α*-syn involved in synucleinopathies may lead to distinct pathological manifestations due to their conformational differences. Therefore, it is crucial to understand, how the surrounding environment influences the conformational properties of *α*-syn and the events that trigger its aggregation and inclusion formation.

### 2.1 Factors potentially contributing to the formation of structural heterogeneity in the conformational strains of *α*-syn

It is well-established that the surrounding conditions of a protein can directly influence its aggregation process, and the impact of factors or co-factors on protein aggregation has been recognized for decades in the study of proteinopathies. For instance, for the tau protein studies, is required to add polyanions, such as heparin, during the *in vitro* experimental conditions to induce protein misfolding, conformational change, aggregation, and further polymerization into filaments. However, by doing so, researchers noticed that the use of these factors culminates in the formation of tau fibrils that are structurally and morphologically heterogeneous (polymorphism of aggregates). More importantly, these are divergent from the filaments isolated from the human brains of patients suffering from tauopathies (Zhang et al., 2019). Similarly, different experimental conditions or factors (such as salt concentration) have been observed to influence the formation of heterogeneous *α*-syn species when studied *in vitro* (Bousset et al., 2013).

As previously mentioned, the propensity of the *α*-syn monomer to misfold and adopt a specific conformation depends on overcoming an energetic barrier, which is influenced by factors and cofactors in the protein’s microenvironment, such as post-translational modifications (PTMs), chaperones, and lipids, that provide the necessary energy for misfolding and aggregation (Goedert, 2001; De Franceschi et al., 2011; Galvagnion et al., 2016; Kam et al., 2018; Candelise et al., 2020; Liu et al., 2021). Additionally, factors such as protein concentration, pH, alternative splicing, mutations in the SNCA gene, metal ions, polyamines (Krasnoslobodtsev et al., 2012; Holmes et al., 2013), proteoglycans (Lashuel et al., 2013), nucleic acids (Hegde and Rao, 2007) and lipids (Fecchio et al., 2018) play crucial roles in this process. A recent hypothesis proposed by Puentes and colleagues suggests that a notable non-protein density observed in high-resolution characterizations of *α*-syn fibrils could be attributed to the negatively charged polymer poly-ADP ribose (PAR). Their use of a proximity ligation assay revealed interactions between PAR and *α*-syn in postmortem brain tissue from PD, PDD, and MSA patients, indicating that these interactions involve positively charged lysine residues in *α*-syn (Puentes et al., 2021). It has been previously shown that the binding of PAR to *α*-syn promotes the rapid aggregation and fibrillation of *α*-syn, inducing cell death (Kam et al., 2018). Also, it is observed that pathological LB inclusions are not composed solely of *α*-syn but also contain various other components such as lipids, the aminoacyl tRNA synthetase complex, synphilin-1, and proteins like Parkin (Ham et al., 2020). Additionally, metal ions, including calcium and iron, are present in these inclusions (González et al., 2019; Shahmoradian et al., 2019; Ivanova et al., 2021). The presence of calcium in LB from PD patients indicates its involvement in the aggregation process and formation of these inclusions (Mattson, 2007). Similarly, iron has been found to accumulate in the substantia nigra and co-deposit with *α*-syn in LB (Zhao et al., 2023). Therefore, a broad range of factors influencing *α*-syn protein’s structure could also explain the heterogeneity of *α*-syn species observed in neurodegenerative diseases.

Factors and cofactors can directly influence the conformational rearrangement of *α*-syn based on their intrinsic biophysical and biochemical properties. This interaction not only offers a potential explanation for the observed polymorphism or conformational strains in *α*-syn aggregates but may also shed light on the initial mechanisms that drive *α*-syn misfolding and aggregation. Understanding these interactions is crucial for comprehending the pathogenesis of synucleinopathies and developing targeted therapeutic strategies to mitigate the harmful effects of *α*-syn aggregation [as recently reviwed in Han et al. (2024)]. Bousset and co-workers provided one of the first evidence demonstrating the impact of the experimental conditions on the formation of *α*-syn aggregates with different toxicity profiles (Bousset et al., 2013). In detail, high salt concentration produced cylindrical aggregates (called fibrils) with a disordered *β*-sheet enriched N-terminal that achieve the nuclei formation (seed that nucleate aggregation pathway) relatively faster. In contrast, low concentration produced planar and twisted structures (ribbons) with a rigid β-sheet enriched N-terminal that takes longer to produce a nucleus during the initial stages of aggregation. This evidence supports the idea that experimental conditions can affect both the rate of aggregate formation and their structure and morphology. Furthermore, the impact of different conformations can be extrapolated to their toxic properties. For example, fibrils produced at high salt concentrations have been shown to induce greater toxicity in human neuroblastoma SH-SY5Y cells (Bousset et al., 2013). When studying toxicity in rats, fibrils and ribbons are the species that induced the greatest toxicity when injected into the substantia nigra. However, the effects at the histological level and observed phenotype were different (Peelaerts et al., 2015), indicating that overall, fibrils showed more severe toxicity compared to oligomers or ribbons; suggesting that different conformations or strains can be differentiated by their cytotoxic effects (Peelaerts et al., 2015). Moreover, the conformations of *α*-syn filaments can differ significantly from the original seed, indicating that seeding products do not necessarily replicate the seed’s atomic structure (Lövestam et al., 2021). Other studies demonstrate that even minor changes in aggregation conditions can result in diverse conformational structures. For instance, amplified aggregates of *α*-syn derived from patients with Lewy body dementia (ampLB) exhibit distinct biological activities *in vitro* compared to human *α*-syn preformed fibrils (hPFF) (Marotta et al., 2021). This observation suggests that the specific conditions under which these structures form are critical. Injection of ampLB into mice expressing human *α*-syn induced pathologies resembling those in LBD patients, yet distinct from those caused by hPFF injection. Additionally, *α*-syn aggregates in ampLB-injected animals retained both the conformational characteristics and biological properties of the original LB-*α*-syn (Uemura et al., 2023).

Monomeric *α*-syn displays remarkable conformational plasticity in its functional monomeric form (Stephens et al., 2019; Stephens et al., 2020). It has been described as a soluble and highly disordered monomer at the cytoplasm of neuronal cells (Theillet et al., 2016; Cattani et al., 2017), but also exists as a lipid-bound monomer (Jao et al., 2008) and as a helical tetramer (Bartels et al., 2011), although the later conformation and its physiological character under normal conditions have been previously largely debated as discussed (Li et al., 2022). Interactions between the positively charged N-terminal domain (region 1–60) and the negatively charged C-terminal domain (96–140) (Takamuku et al., 2022) determine the morphology of monomeric *α*-syn conformations. In detail, electrostatic and hydrophobic interactions lead to partial folding of *α*-syn. They are important to protect the non-amyloid-β component (NAC) (region 61–95) from interacting with other molecules, such as proteins, as NAC acts as a hydrophobic core responsible for aggregation (Stephens et al., 2018; Stephens et al., 2019; Stephens et al., 2020; Kasen et al., 2022; Privat et al., 2022). For instance, upon the interaction between N and C-terminal *α*-syn adopts a protective –hairpin- conformation (Ubbiali et al., 2022), which under certain conditions, can be changed into a more extended, flexible, and disordered structure (Ubbiali et al., 2022; Hou et al., 2023). A structural vulnerability to aggregation has been reported to be directly linked to the exposure of the N-terminal and the beginning of the NAC region to the surrounding environment (Stephens et al., 2020). Changes in the net surface charge of *α*-syn, following, e.g., pH, metal ions, salt ions, polyamines, and others can lead to an exposure of the NAC domain by enhancing side chain repulsion or by shielding the N and C-terminals charged, allowing for more energetically favorable packing of fibrils (Binolfi et al., 2006; Buell et al., 2014; Roeters et al., 2017).

All familial mutations in the SNCA gene linked to PD are located within the N-terminal domain, suggesting that these amino acid substitutions can induce structural rearrangements, potentially promoting aggregation. For instance, A53T, A53E, or A30P, have been reported to display a propensity for aggregation and further fibril formation (Lashuel et al., 2002; Ulrih et al., 2008; Afitska et al., 2017; Bengoa-Vergniory et al., 2017; Landeck et al., 2020; Mahul-Mellier et al., 2020). E46K mutation alters the salt bridge promoting aggregation and reorganizing the fibrillar structure to accelerate aggregation (Zhao et al., 2020; Long et al., 2021). H50Q mutation reduces the solubility of *α*-syn, resulting in accelerated aggregation and fibril formation *in vitro* (Appel-Cresswell et al., 2013; Ghosh et al., 2013; Porcari et al., 2015). As mentioned before, the intermolecular interactions between the N and C-terminal domains of *α*-syn are important in modulating aggregation propensity (Stephens et al., 2018; Stephens et al., 2019). Therefore, mutation in key regions with C-terminal interaction, such as calcium-binding regions, can result in the conformational change that leaves the N-terminal of *α*-syn exposed to solvents in the surrounding environment (Stephens et al., 2019; Stephens et al., 2020). Overall, different mutations can lead to different levels of *α*-syn exposure, differences in stabilization, and differences in perturbation of the conformation ensemble (Bertoncini et al., 2005; Wise-Scira et al., 2013; Flagmeier et al., 2016; Bhattacharyya et al., 2018; Stephens et al., 2020; Xu et al., 2022; Xu et al., 2023). Furthermore, it has been suggested that the deletion of one or more exons due to alternative splicing in the *α*-syn gene (SNCA) could have an impact on the aggregation propensity (Röntgen et al., 2024). This alternative splicing results in the expression of four *α*-syn isoforms [as reviewed in Beyer and Ariza (2013) and Gámez-Valero and Beyer (2018)]. The most abundant is the 140-amino acid protein (*α*-syn-140) which we generally refer to in this review. The other three isoforms are *α*-syn-126, *α*-syn-112, and *α*-syn-98 generated by deletion of exon 3 (amino acids 41–54), exon 5 (amino acids 103–130), or both exons, respectively [as reviewed in Beyer and Ariza (2013); Gámez-Valero and Beyer (2018)]. Interestingly, it has been suggested that splicing isoforms are expressed differently in different synucleinopathies (Beyer et al., 2008a; Beyer et al., 2008b; Beyer and Ariza, 2013). Despite the evidence, their impact at the biological and pathological level has not yet been elucidated and the need for further research becomes evident.

The differences in functional and pathological effects of the isoforms could be determined by their structural arrangement and interactions. For instance, deletion of exon 3 that leads to the formation of *α*-syn-126 isoform may also alter the N-terminal protein-membrane interaction domain, potentially impacting aggregation propensity (Röntgen et al., 2024). For instance, some authors have highlighted the potential for inducing *α*-syn aggregation in the presence of lipids due to the deletion of exon 3 (Röntgen et al., 2024). It has been shown that familial mutations in this region are influenced by lipid-induced aggregation (Flagmeier et al., 2016). Conversely, the deletion of exon 5, in the isoform, could result in a greater propensity for aggregation, induced by the significant shortening of the C-terminal domain (Beyer, 2006; Röntgen et al., 2024). Both *α*-syn-112 and *α*-syn-98 isoforms have been shown to produce accelerated aggregation compared to *α*-syn-140 when studied *in vitro* (Röntgen et al., 2024). In addition, these isoforms produce distinct aggregate morphologies (dense agglomerations of fibrils), as demonstrated by Transmission Electron Microscopy (TEM) (Röntgen et al., 2024). Interestingly, different transcripts have been shown in disease brains (Beyer, 2006; Beyer et al., 2008a; Beyer and Ariza, 2013; Cardo et al., 2014). For instance, the *α*-syn-112 isoform has been reported to be highly increased, while the expression levels of the *α*-syn-126 isoform are much lower in the prefrontal cortex of DLB patients (Beyer et al., 2008a). Interestingly, this isoform is increased in the frontal region of PD brains, while no significant differences are observed in multiple system atrophy (MSA) patients (Beyer et al., 2008a). The *α*-syn-98 isoform, characterized by the absence of exons 3 and 5, is a brain-specific splice variant with varying expression levels in different regions of the fetal and adult brain. For instance, in the frontal cortices of patients with DLB, PD and MSA compared to controls (Beyer et al., 2008b). Additionally, post-translational modifications can affect *α*-syn properties. For instance, glycation of the N-terminal region affects aggregation and toxicity by disrupting its binding to lipid membranes, which in turn impairs the proteasomal pathway (Dikiy and Eliezer, 2012). Similarly, C-terminal truncation not only increases aggregation propensity by exposing the NAC region (Li et al., 2005; Sorrentino et al., 2018; Wang et al., 2016), but also leads to alterations in the lysosomal pathway. Truncation affects the negative charge leading to a decrease in electrostatic repulsion allowing more energetically favorable aggregation (Stephens et al., 2018). Also, it has been reported that the C-terminal truncation is prone to induce mitochondrial dysfunction (Games et al., 2013) and alterations in the lysosomal pathway (Krzystek et al., 2021). Other modifications such as *α*-syn nitration (Giasson et al., 2000; He et al., 2019) can have serious implications for disease, as this modification are markers for oxidative and nitrative damage (He et al., 2019). It has been observed that monomeric or dimeric nitrated *α*-syn triggers fibril formation by recruiting unmodified *α*-syn, whereas oligomeric *α*-syn (induced by nitration) appears to block fibril formation (Hodara et al., 2004). This suggests that the nitrated oligomeric state could be important in the propensity for rapid aggregation and accelerated pathogenesis of PD (Hodara et al., 2004). It has been also suggested that other modification such as *α*-syn SUMOylation could have enhancing and suppressing roles on *α*-syn aggregation (Yoo et al., 2022). However, the consensus is that *α*-syn SUMOylation positively impact *α*-syn pathological outcome by enhancing *α*-syn degradation and preventing fibril polymerization (Krumova et al., 2011; Rott et al., 2017).

Apart from genetic factors, some environmental conditions promote *α*-syn aggregation, including polyvalent cations such as polyamines, proteoglycans, and PAR. For instance, polyamine spermine can induce a conformational change in *α*-syn, initiating the aggregation process through dimerization (Holmes et al., 2013). A combination of cationic charge and length of aliphatic chains that separate the amino groups may also impact the heterogeneity of *α*-syn oligomerization (Krasnoslobodtsev et al., 2012). In addition, it has been reported that upon interaction with various metal ions, *α*-syn changes its conformation (Binolfi et al., 2006). Studies have demonstrated that the highly negatively charged C-terminal domain of *α*-synuclein binds calcium, which in turn enhances the protein’s lipid-binding capacity (Emamzadeh, 2016; Lautenschläger et al., 2018). Calcium binding induces a conformational rearrangement in *α*-syn, exposing the NAC core and promoting hydrophobic interactions with adjacent molecules (Giasson et al., 2001). This structural change facilitates the binding of *α*-syn to lipid membranes and vesicles, potentially driving filament formation (El-Agnaf et al., 1998). This process is considered a crucial initial step in the oligomerization and subsequent polymerization of *α*-syn (Tsigelny et al., 2012; Miraglia et al., 2018; Ugalde et al., 2019; Ugalde et al., 2020; Choi et al., 2022). Certain modifications to the N-terminal domain of *α*-syn can impact its ability to bind to membranes and vesicles (Dikiy and Eliezer, 2012), potentially disrupting its interaction with lipid surfaces (Dikiy and Eliezer, 2012). Other metals have been reported to have an important impact on *α*-syn aggregation. Zhao and coworkers characterized the structural interaction between Fe3+ and *α*-syn in both monomeric and *α*-syn filaments, showing that Fe3+ (at a low molar ratio) can promote rapid *α*-syn fibril formation, whereas at high concentration of Fe3+ fibril formation is inhibited. Based on the ultrastructural data, the authors described that Fe3+ can directly bind to the *α*-syn fibril, through the negatively charged binding pocket formed by His50 and Glu57 on the fibril surface (Zhao et al., 2023). They further validate their results by testing the effect of mutation of His50, which abolishes the Fe3 + facilitated fibrillation of *α*-syn (Zhao et al., 2023).

In addition, interaction with other proteins, such as with PrPc, tau, and others, can cause cross-seeding and promote rapid *α*-syn aggregation (Katorcha et al., 2017; Bassil et al., 2020). This body of evidence suggests crosstalk between different proteins relevant in proteinopathies, promoting co-pathological conditions (Bassil et al., 2020; Bassil et al., 2021). This shows that seed-producing and pathologically relevant aggregated proteins can modulate the pathology of other proteins, for example, *α*-syn in the modulation of tau pathology. Further evidence for this comes from studies demonstrating co-pathology with overlapping Aβ plaques, tau tangles and *α*-syn characteristics in brains from patients with AD, PD and PDD (Colom-Cadena et al., 2013; Irwin et al., 2017; Robinson et al., 2018), although much remains to be elucidated about the consequences of primary pathological proteins on secondary, subsequently triggered, pathology. Overall, substantial evidence suggests that various factors and co-factors significantly influence *α*-syn conformational changes and aggregation. These effects are likely dependent on the specific biochemical and biophysical properties of each factor, which interact closely with the vulnerable *α*-syn protein.

## 3 Liquid–liquid phase separation underlying the pathological cascade of Parkinson’s disease

Increasing evidence highlights that oligomers and protofilaments are the most neurotoxic species responsible for dopaminergic neurodegeneration in PD (Sharon et al., 2003; Karpinar et al., 2009; Cremades et al., 2017; Gadhe et al., 2022). To unravel the mechanisms behind *α*-syn pathology in neurodegeneration, it is crucial to comprehend the events that initiate the transformation of monomeric *α*-syn into its pathological forms. However, the key processes driving this transformation, and the subsequent onset of pathology remain poorly understood. Recent emerging evidence suggests that a phenomenon called liquid–liquid phase separation (LLPS) occurs as an early event preceding the aggregation of *α*-syn associated with neurodegeneration (Elbaum-Garfinkle, 2019; Ray et al., 2020; Zbinden et al., 2020). LLPS is a physiological, spontaneous, and reversible process through which various components, such as proteins and nucleic acids, organize into membrane-less bodies resembling liquids, known as biomolecular condensates (Banani et al., 2016; Wegmann et al., 2018). LLPS enables the formation of distinct compartments that are isolated from the surrounding environment, facilitating specialized functions and biochemical reactions within these compartments (Banani et al., 2016; Boeynaems et al., 2018; Alberti et al., 2019; Wang et al., 2021; Gouveia et al., 2022; Hou et al., 2023). Although LLPS formation has been described under normal physiological conditions, this phenomenon has also been observed in relevant neurodegenerative disorders (Wegmann et al., 2018; Borcherds et al., 2021; Darling and Shorter, 2021; Hardenberg et al., 2021; Kanaan et al., 2020; Ray et al., 2020; Sprunger and Jackrel, 2021; Ziaunys et al., 2024). For this reason, the formation of LLPS has recently been proposed as a mechanism potentially underlying PD and other neurodegenerative diseases (Hou et al., 2023; Mukherjee et al., 2023; Han et al., 2024).

**Figure 4:**
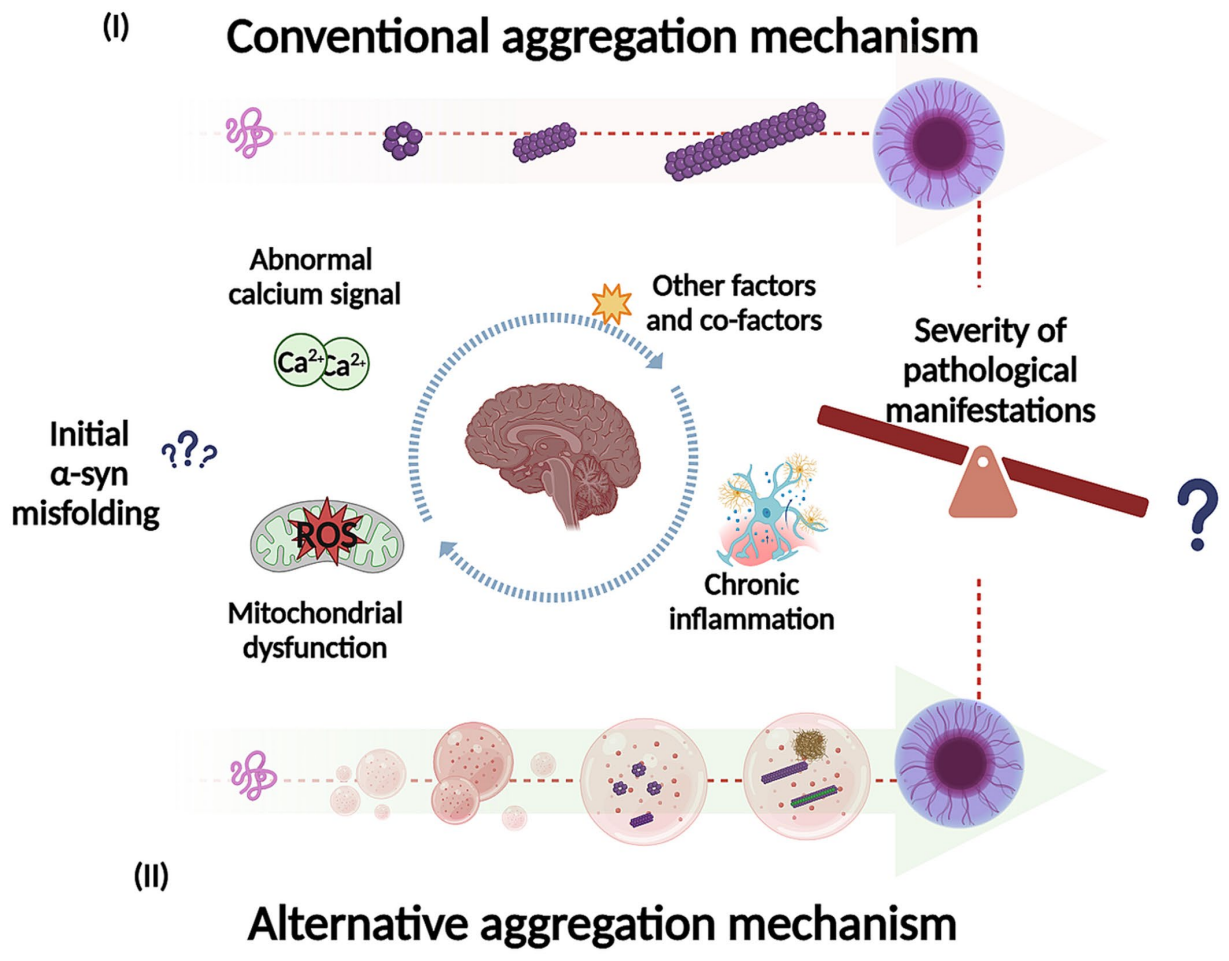
Representation of potential mechanisms for *α*-syn self-assembly and aggregation into intermediate species, fibrils and inclusions. In the conventional aggregation mechanism (I), monomers could self-assemble into dimers and then progressively into different oligomeric species on pathway to further form filaments, fibrils, and inclusions. In addition, the alternative aggregation mechanism (II), *α*-syn could undergo liquid–liquid phase separation (LLPS) to form droplets and transition to liquid-to-solid phase, which could potentially undergo gel-like structure (hydrogel) with embedded oligomers and filaments. The LLPS and condensate formation could potentially be key to understand what trigger *α*-syn misfolding and conformational changes (influenced by environmental conditions). In addition, this process may play an important role in the pathological manifestations as well in its severity. Created with BioRender.com

Studies have shown that soluble *α*-syn can undergo LLPS to form droplets, which result from interactions between its negatively charged regions and positively charged molecules in the environment (Mukherjee et al., 2023). These droplets might act as intermediates in the transition from native *α*-syn to its aggregated disease-associated form. Over time, the droplets can undergo a liquid-to-solid phase transition, eventually forming a gel-like structure with embedded oligomers and filaments (Ray et al., 2020; Mukherjee et al., 2023; Han et al., 2024; Ziaunys et al., 2024). LLPS and condensate formation have been proposed as an alternative aggregation mechanism and may potentially underlie the basis of neurodegenerative diseases (Figure 4) (Hou et al., 2023; Mukherjee et al., 2023; Han et al., 2024). Furthermore, this alternative mechanism could be key to understand the factors that trigger *α*-syn misfolding and conformational changes with the development of pathology, influenced by environmental conditions. Specifically, this aggregation pathway is affected by the crowded nature of the cellular environment (Mukherjee et al., 2023). Additionally, different aggregation routes can explain the diverse conformational strains of *α*-syn, which in turn may account for the varying clinical presentations of PD and other synucleinopathies (Lashuel et al., 2013; Koga et al., 2021). Recent research supports this hypothesis by comparing *α*-syn aggregates formed under conditions that either promote or inhibit LLPS (Ziaunys et al., 2024). A study found that when LLPS was encouraged, using a crowding agent and high protein concentrations, *α*-syn formed a diverse range of structures with various shapes and secondary structures. Conversely, in conditions that inhibit LLPS, such as low protein concentration and no crowding agents, different aggregate forms were observed (Ziaunys et al., 2024). The authors highlight the relevance that this could have in the context of LLPs formed *in vivo*, given the abundance and rapid formation of these aggregates. This process may play an important role in both the onset and progression of these disorders (Ziaunys et al., 2024) which can be extrapolated to the severity of the pathological manifestations. Furthermore, some of these structures could have greater toxicity and more efficiently catalyze the conversion of native *α*-syn protein, compared to aggregates formed through a different pathway. Additionally, this study suggests that the formation of LLPS and condensate could be a critical event in the appearance of conformational strains. Another research team developed a cell model to explore how fibrillary seeds interact with synthetic *α*-syn condensates, shedding light on their role in driving prion-like disease progression (Piroska et al., 2023). Interestingly, the presence of pre-formed condensates prompts the transition of *α*-syn (exogenous fibrillary seeds) from a liquid to a solid-like state through phase separation, resulting in the formation of needle-shaped amyloid structures within the condensates in cells (Piroska et al., 2023). This finding supports the idea that *α*-syn condensates may play a crucial role in initiating pathology and potentially drive its subsequent spread. Additionally, the study observed that different strains of *α*-syn fibrils lead to a remodeling of these condensates over time (Piroska et al., 2023).

As discussed previously, electrostatic, and hydrophobic interactions are important in the aggregation process, likewise, these interactions can also modulate the formation of LLPS (Gallardo et al., 2020; Sawner et al., 2021; Huang et al., 2022). For example, metal ions can influence the progression of *α*-syn phase separation, while this process can be reversed by metal ion chelators (Ray et al., 2020; Sawner et al., 2021; Huang et al., 2022; Xu et al., 2022b). Similarly, *α*-syn protein modifications, such as A53T and E46K mutations, may contribute to the modulation of *α*-syn LLPS and fibril formation (Ray et al., 2020). PTMs also mediate *α*-syn LLPS modulation. For example, S129 phosphorylation accelerates *α*-syn LLPS and its amyloid transition (Ray et al., 2020; Hou et al., 2023). In contrast, N-terminal acetylation increases protein solubility and delays *α*-syn LLPS (Ray et al., 2020). Binding to negatively charged lipids promotes the folding of *α*-syn into a tighter conformation leading to the rapid formation of filaments (Fusco et al., 2017) and LLPS. Although the *α*-syn LLPS formation has been recently established as a potential factor in pathology, the mechanism underlying its toxicity remains unknown. It has recently been proposed that under physiological conditions droplets could be highly regulated by cellular machinery. In contrast, alterations in proteostasis in an environment appropriate for the development of the disease could trigger irreversible and aberrant phase separation that leads to subsequent aggregation and pathology (Hou et al., 2023).

In the next section, we will now attempt to briefly explore the potential relationship between *α*-syn, the LLPS phenomenon, and relevant cellular processes such as mitophagy regulation, which plays an essential role in the pathogenesis of PD.

### 3.1 Potential crosstalk between *α*-syn, LLPS formation, and cellular deregulation

As previously described, it has been hypothesized that cellular stress and pathological conditions related to diseases, such as PD-related factors, can alter the LLPS properties (dynamics and reversibility) leading to aberrant LLPS-assembled structures (such a filaments) that can elicit toxicity (Darling and Shorter, 2021). LLPS formation is very sensitive to perturbations on both the protein and cellular environment. Thus, disease-relevant mutations and factors that promote or increase *α*-syn susceptibility to aggregation are also important in *α*-syn LLPS dysregulation. Regarding the cellular machinery relevant to PD, a functional relationship has been established between *α*-syn, chronic neuroinflammation, immune dysregulation, and mitochondrial dynamics. This relationship is widely recognized in the pathology of PD. However, the underlying mechanisms involved in these interactions remain to be investigated.

Alterations such as cellular stress, *α*-syn aggregation and LLPS formation could be important aspects in the modulation of pathology. In line with this, since LLPS formation is widely present in aberrant *α*-syn aggregates as well as in some physiological processes of proteins related to elimination of damaged mitochondria (mitophagy) such as PINK1 (PTEN-induced kinase 1)/Parkin (an E3 ubiquitin ligase). For this, recent attention has been paid to understanding the aspects associated with LLPS transformation and pathology, in the context of the regulation of mitophagy processes (Noda et al., 2020; Yamasaki et al., 2020; Peng et al., 2021; Xing et al., 2021) and aberrant transformation of both *α*-syn and LLPS [as reviewed in Hou et al. (2023)]. Accordingly, *α*-syn-induced mitochondrial impairment is recognized as a key factor contributing to neurodegenerative pathogenesis. Therefore, the efficient elimination of impaired mitochondria is essential for neuronal protection and subsequent degeneration. Mitophagy is the main pathway for its selective removal and protects against mitochondrial dysfunction and its associated cytotoxicity (Liu et al., 2019; Malpartida et al., 2021). Thus, a link between the regulators of mitophagy, PINK1/Parkin and *α*-syn-induced mitochondrial alterations has been widely related to PD. In line with this, dysfunction, or loss of either PINK1 or Parkin aggravates the characteristic phenotypes induced by pathological *α*-syn (Stichel et al., 2007; Gispert et al., 2015; Creed and Goldberg, 2018; Creed and Goldberg, 2020). While overexpression can improve these phenotypes (Todd and Staveley, 2008; Chung et al., 2020; Krzystek et al., 2021; Wilkaniec et al., 2021).

Our research group has shown that cellular exposure to exogenous *α*-syn fibrils or oligomers reduces Parkin protein level along with mitochondrial damage (Wilkaniec et al., 2019; Wilkaniec et al., 2021). In addition, it has been shown that exposure to exogenous *α*-syn oligomers induces oxidative and nitrosative stress, resulting in post-translational modifications of Parkin, leading to its autoubiquitination and degradation (Yao et al., 2004; Kazmierczak et al., 2008; Wilkaniec et al., 2019). Thus, it has been established that abnormal *α*-syn induces Parkin downregulation, leading to exacerbated mitochondrial damage through a negative feedback loop between pathological *α*-syn and Parkin (Jęśko et al., 2019). Relatively recent evidence suggests that Parkin inactivation or dysfunction might not only impact autophagy but also the production of aberrant LLPS (Brahmachari et al., 2019; Hou et al., 2023; Kang et al., 2023). LLPS formation has been described in several steps during the autophagy process (Bjørkøy et al., 2005; Bhattacharya and Behrends, 2020; Fujioka et al., 2020; Noda et al., 2020) and could be an important regulator of autophagic degradation of protein aggregates (Zhang, 2022). During mitophagy on damaged mitochondria, PINK1 activates Parkin which physically interacts with various ubiquitin-coupled enzymes (E2 enzymes) (such as UBE2L3 and UBE2D) (Hayashida et al., 2023) and only then Parkin forms LLPS inside the cell (Hayashida et al., 2023 and reviewed in Hou et al., 2023). This process activates the ubiquitin ligase family (E3) (RING-between-RING type E3 ligases) and subsequently the ubiquitination of substrate proteins (Hayashida et al., 2023). Structurally, the IBR region is the key domain in Parkin for phase separation and condensation formation (Hayashida et al., 2023). Despite this, it is far from clear whether Parkin formation of LLPS can be affected after PINK1 removal during PD disease. Therefore, further studies are needed to understand the role of PINK1/Parkin in the context of aberrant LLPS and impaired mitophagy.

Parkin-interacting substrate (PARIS) has been reported as a potential modulator of LLPS in neurodegenerative diseases, due to its ability to undergo LLPS and form solid amorphous structures (Kang et al., 2023). It has been hypothesized that PARIS accumulation could inhibit important signaling pathways contributing to mitochondrial dysfunction and dopaminergic neuronal death (Scarffe et al., 2014; Siddiqui et al., 2015; Siddiqui et al., 2016; Stevens et al., 2015; Brahmachari et al., 2019; Jo et al., 2021; Kumar and Kumar, 2019; Pirooznia et al., 2020). Parkin plays a fundamental role in this scenario, since its inactivation can contribute significantly to the accumulation of PARIS due to its inhibition of proteasomal degradation (Brahmachari et al., 2019; Kang et al., 2023). PARIS accumulation led to an imbalance of energy metabolism because of repression of peroxisome proliferator-activated receptor gamma coactivator 1-alpha (PGC-1*α*) (Stevens et al., 2015). In addition, PARIS accumulation has been shown to result in overactivation of PAR (Kang et al., 2023). These events can have detrimental results as poly (ADP-ribose) (PAR) can get attached to the C-terminal region of PARIS enhancing its LLPS and solidification leading to insoluble aggregate formation. This aggregate can then sequester PGC-1α potentially contributing to PD pathology (Kang et al., 2023).

It has been reported that alterations such as factors or events involved in the development and progression of the disease, such as DNA damage or physiological stress, can induce an increase in PAR levels (Altmeyer et al., 2015; McGurk et al., 2018; Patel et al., 2015). There is evidence showing that PAR can induce LLPS formation from intrinsically disordered region (IDR)-containing proteins (Altmeyer et al., 2015; McGurk et al., 2018; Patel et al., 2015) such as *α*-syn. Interestingly, it has been observed that multifunctional protein 2 (AIMP2) that interacts with the abnormal *α*-syn and aminoacyl tRNA synthase complex can activate poly (ADP-ribose) polymerase-1 (PARP-1), which in turn produces PAR (Kam et al., 2018) contributing to dopaminergic neuronal death (Berger et al., 2018; Kam et al., 2018). This suggests a link between Parkin inactivation, PARIS accumulation and abnormal *α*-syn in feedback that may aggravate the disease. Brahmachari and collaborators demonstrated that exposure to preformed *α*-syn fibrils (PFF) leads to the phosphorylation and inactivation of Parkin, resulting in the accumulation of PARIS in a Parkinson’s disease mouse model (Brahmachari et al., 2019). Furthermore, it has been observed that injection of *α*-syn PFF positively regulates PARIS and PAR levels (Kam et al., 2018; Brahmachari et al., 2019) resulting in high molecular weight PARIS species. In addition, Kang et al. demonstrated in a Parkinson’s disease mouse model that the injection of *α*-syn PFF triggers the activation of PAR production (Kang et al., 2023). The authors reported that PAR induces the conversion of *α*-syn species into a more toxic strain, aggravating neurodegeneration. In general, PAR appears to cause SDS-resistant PARIS aggregates. And the appearance of the insoluble high molecular weight PARIS species was only prevented by deletion of PARP-1, suggesting that PAR may lead to LLPS- and PAR-mediated PARIS solidification in this animal model (Kang et al., 2023). PARIS solidification was observed in the brains of *α*-syn PFF-injected mice and adult Parkin knockout (KO) mice, but not in PARP-1-deficient mice injected with *α*-syn PFF. This finding demonstrates that PARIS undergoes LLPS- and PAR-mediated solidification (Kang et al., 2023). Overall, these studies underscore the importance of understanding the mechanisms underlying phase separation and the transition of LLPS in both physiological and pathological contexts for therapeutic development. Additionally, it would be of great interest to further investigate how different conformations of *α*-syn aggregates could impact the LLPS process in relation to mitophagy and other relevant cellular processes. This also highlights the significance of studying cellular conditions, factors, and co-factors that may influence aberrant LLPS and *α*-syn physiology.

## 4 Potential therapeutic strategies to address synucleinopathies

A variety of studies have explored strategies targeting *α*-syn to treat human synucleinopathies (McFarthing et al., 2023; Price et al., 2018; Wagner et al., 2013; Perni et al., 2017; Staats et al., 2020; Chia et al., 2023), highlighting their potential to generate encouraging results. Thus, *α*-syn-targeting therapies based on active immunization (Poewe et al., 2021; Volc et al., 2020; Eijsvogel et al., 2024), passive immunization (Lang et al., 2022; Pagano et al., 2022) as well as the use of *α*-syn aggregation inhibitors (Wagner et al., 2013; Smit et al., 2022; Prince et al., 2023) have achieved promising results in recent years. Despite this, their clinical relevance remains to be determined [as reviewed in Calabresi et al. (2023)]. So far, treatment with a small molecule called Minzasolmin (UCB0599) stands out as a potential disease-modifying therapy, which reduces the formation of *α*-syn aggregates (as oligomers) (Wagner et al., 2013; Smit et al., 2022; Prince et al., 2023). This treatment is currently being tested in phase 2 clinical trials in patients with early PD, and its clinical efficacy is expected to be published in the upcoming years.

While these strategies clearly have the potential to treat human synucleinopathies, their use is still limited, and significant obstacles remain, given the complexity of the disease itself [as reviewed in Calabresi et al. (2023), McFarthing et al. (2023), and Otzen (2024)]. A potential limitation, for instance, is that the mechanisms of action of certain therapies may lack full specificity for the relevant pathogenic *α*-syn species due to the extensive heterogeneity of aggregates and diverse conformational strains in which *α*-syn can polymerize. This variability complicates the development of highly specific therapeutic approaches, making it challenging to target a single pathogenic species precisely. Therefore, recognizing the disease-relevant conformation of *α*-syn (atomic conformation) with high specificity could represent a new opportunity for developing methods that accurately replicate the relevant strains causing human diseases in the appropriate study models. This unlocks a new paradigm for identifying specifically designed and personalized therapies to interfere with developing and propagating disease-specific *α*-syn aggregates in the brain. In addition, it provides the foundation to develop methods to recognize individual *α*-syn strains in a personalized manner, which could improve the stratification of patient populations for clinical trials and the development of efficient diagnostic tools.

Another challenge to overcome is the chemical modification to which *α*-syn is exposed, which can impact *α*-syn’s biochemical characteristics and biological activities. Since *α*-syn can interact with other molecules, some may cause negative effects (such as interaction with other proteins, membranes, and other molecules). Despite this, understanding the mechanisms underlying *α*-syn conformational modifications upon interactions with the surrounding environment could also lead to opportunities to inhibit and neutralize *α*-syn aggregation and fibril formation or even generate alternative species with less toxicity (Han et al., 2024). For example, research has shown that the post-translational modification of *α*-syn monomers with O-GlcNAc leads to the formation of *α*-syn amyloid-like fibrils [called *α*-syn (gS87)], which possess distinct structural characteristics that significantly diminish their seeding potential in both *in vitro* and rodent PD models (Balana et al., 2024). This finding opens up new avenues for exploring innovative therapeutic strategies for treating synucleinopathies, highlighting the potential for targeted interventions that leverage these unique modifications.

A more comprehensive understanding of *α*-syn in the context of pathology, along with the challenges associated with developing effective therapies, has stimulated the exploration of new aggregation mechanisms, such as condensation (Mitrea et al., 2022; Vendruscolo and Fuxreiter, 2022; Han et al., 2024). Consequently, approaches previously employed to identify small molecules that stabilize and inhibit *α*-syn aggregation via conventional mechanisms can now be adapted to enhance stability through the condensation pathway (Xu et al., 2022a; Dada et al., 2024; Li et al., 2024). As an example, a recent study reported that the aminosterol claramine could stabilize *α*-syn condensates as well as inhibit the primary nucleation of *α*-syn within the condensates, both *in vitro* and in a *Caenorhabditis elegans* PD model (Dada et al., 2024). This is especially important considering that LLPS formation could be the mechanism potentially underlying PD and other neurodegenerative diseases. Thus, additional studies exploring this approach would be very informative and may significantly impact drug development.

Growing evidence supports the promising and valuable potential of targeting *α*-syn to develop clinically effective therapeutic strategies. However, it also highlights the importance of thoroughly understanding the mechanisms underlying *α*-syn conformational modifications and their interactions with the surrounding environment.

## 5 Summary

Evidence suggests that intermediate species of *α*-syn aggregates are responsible for cell death in PD. However, the molecular events involved in *α*-syn aggregation and its relationship with disease onset and progression are not fully understood. However, the molecular events involved in *α*-syn aggregation and their relationship to disease initiation and progression are not fully understood. Current research is exploring LLPS and condensate formation, which have been proposed as alternative mechanisms that might not only underlie *α*-syn pathology but also contribute to heterogeneity in synucleinopathies. Cellular stress and pathological conditions may affect *α*-syn monomeric structure, aggregation, and LLPS behavior, potentially leading to the formation of aberrant structures within LLPS and condensates that result in toxicity. Therefore, it is reasonable to hypothesize that these factors may influence the dynamic interactions of monomer conformers and affect specific aggregation pathways, which could have a significant impact on the onset and progression of the disease.
